# Water Deficit Affected Flavonoid Accumulation by Regulating Hormone Metabolism in *Scutellaria baicalensis* Georgi Roots

**DOI:** 10.1371/journal.pone.0042946

**Published:** 2012-10-15

**Authors:** Yuan Yuan, Yunjun Liu, Chong Wu, Shunqin Chen, Zhouyong Wang, Zhaochun Yang, Shuangshuang Qin, Luqi Huang

**Affiliations:** 1 Institute of Chinese Material Medica, Academy of Chinese Medical Sciences, Beijing, China; 2 Institute of Crop Science, Chinese Academy of Agricultural Sciences, Beijing, China; National Taiwan University, Taiwan

## Abstract

The content of flavonoids especially baicalin and baicalein determined the medical quality of *Scutellaria baicalensis* which is a Chinese traditional medicinal plant. Here, we investigated the mechanism responsible for the content and composition of flavonoids in *S. baicalensis* under water deficit condition. The transcription levels of several genes which are involved in flavonoid biosynthesis were stimulated by water deficit. Under water deficit condition, fifteen up-regulated proteins, three down-regulated proteins and other six proteins were detected by proteomic analysis. The identified proteins include three gibberellin (GA)- or indoleacetic acid (IAA)-related proteins. Decreased endogenous GAs level and increased IAA level were observed in leaves of *S. baicalensis* which was treated with water deficit. Exogenous application of GA or α-naphthalene acelic acid (NAA) to plants grown under water deficit conditions led to the increase of endogenous GAs and the decrease of IAA and flavonoids, respectively. When the synthesis pathway of GA or IAA in plants was inhibited by application with the inhibitors, flavonoid levels were recovered. These results indicate that water deficit affected flavonoid accumulation might through regulating hormone metabolism in *S. baicalensis* Georgi.

## Introduction

Flavonoids are important plant secondary metabolites which have important effect on plant physiology [1]. Plant flavonoids exhibit several medicinal properties, such as antioxidant activity and anti-inflammatory activity [2], and these flavonoids largely determine the quality of medicinal plants. For example, flavonoids are regarded as one of the most important determinants of quality in red grapes and wine [3]. Various biotic and abiotic stress conditions also affected the accumulation of flavonoids in plant vegetative tissues and organs [4].

The roots of *Scutellaria baicalensis* Georgi are used to treat various diseases in Chinese traditional medicine. The active compounds of *S. baicalensis* include baicalin, baicalein, wogonoside, wogonin, neobaicalein, visidulin I, and oroxylin A, and these compounds exhibit anti-inflammatory, anti-tumor, and anti-HIV activities [5]. These flavonoids, especially baicalin and baicalein, are regarded as the most important determinants of the quality of *S. baicalensis* [6]. Baicalin is synthesized via the phenylpropanoid pathway by the activities of several enzymes, including phenylalanine ammonia-lyase (PAL), cinnamate 4-hydroxylase, 4-coumarate:CoA ligase, chalcone synthase (CHS), and chalcone isomerase [7]. β-glucuronidase (GUS) catalyze baicalin to baicalein which then can be catalyzes to 6,7-dehydrobaicalein by peroxidase (POD), detoxifying H_2_O_2_ to water [8], [9]. Baicalein can be catalyzed back to baicalin by UDP-glucuronate: baicalein 7-O-glucuronosyltransferase (UBGAT) [10].

Water deficit affects flavonoid biosynthesis in plants. Vincent et al. [11] found the lower lignin levels in leaves of plants treated with water deficit, compared with those well-watered plants. It has also been found that drought treatment strongly upregulated the anthocyanin biosynthesis in ripening fruit [12]. Regulated deficit irrigation can be used to improve berry and wine quality, because water deficit early in the season can lead to more anthocyanins and phenolics [13], [14]. Water deficit can also enhance the flavonoid production in cell suspension culture of *Glycyrrhiza inflata* Batal [15]. Guidi et al. [16] reported that antioxidant phenylpropanoid concentrations increased in response to water stress in shade leaves. In a previous study, we found that light conditions could affect the expression of *GUS* and *UBGAT*, and the baicalein∶baicalin ratio by influencing the flavonoid metabolism and the conver between individual flavonoids [17].

Flavonoid biosynthesis is also affected by plant hormones which regulating the plant growth and development. Anthocyanin accumulation could be enhanced by abscisic acid (ABA) treatment and suppressed by application of synthetic auxins or 1-naphthaleneacetic acid (NAA) [18]–[20]. Flavonoid compounds have been shown to modulate the transport of phytohormone auxin [21], [22]. Besseau et al. [23] reported that flavonoid accumulation affects auxin transport and plant growth in Arabidopsis.

Our previous work revealed that low levels of rainfall are closely associated with baicalein content in 19 production areas [24], indicating that water status is an important factor affecting the flavonoid mechanisms that in turn determine the quality of *S. baicalensis*. Here, we investigated the mechanism responsible for the content and composition of flavonoids in *S. baicalensis* grown under water deficit conditions.

## Results

### Water deficit affected the flavonoid accumulation

**Figure 1:**
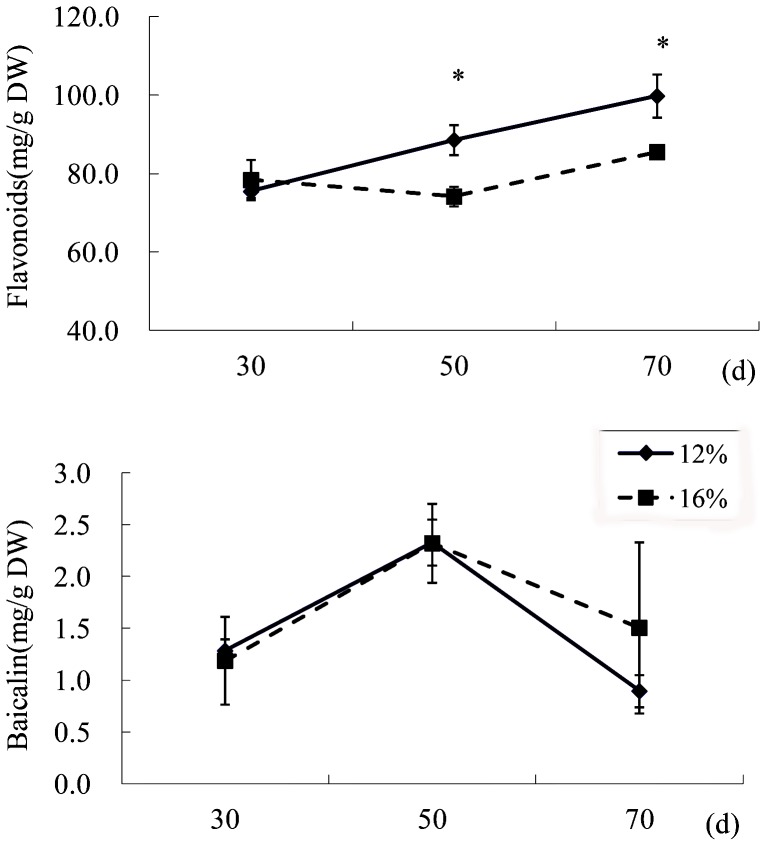
Effects of water deficit on flavonoid accumulation in *S. baicalensis*. Total flavonoids and baicalin in the leaves of *S. baicalensis* grown under 16% SWC as a control (broken line) and 12% SWC as a water deficit treatment (solid line). Vertical bars indicate the standard deviation of three biological replicates. Asterisks indicate a significant difference at the *P*<0.05 level.

*S. baicalensis* plants grow in the north of China where 15–20% of soil water content (SWC) is usually suitable for crop growth, whereas 12–15% SWC and 8–12% SWC is considered as mild and moderate drought stress, respectively (http://www.natesc.gov.cn). In this study, three-month-old plants which have grown under well-water condition were then kept SWC as 12% SWC (water deficit) or 16% SWC (control) condition. Water deficit significantly increased the total flavonoid contents both in roots [25] and in leaves at 50 d and 70 d (Figure 1), whereas the content of baicalin did not change much in leaves (Figure 1). HPLC analysis revealed that the major active compounds were baicalin and baicalein, and that these compounds mainly accumulated in roots (Data not shown).

### Water deficit increased the expression of several flavonoid biosynthesis genes

**Figure 2:**
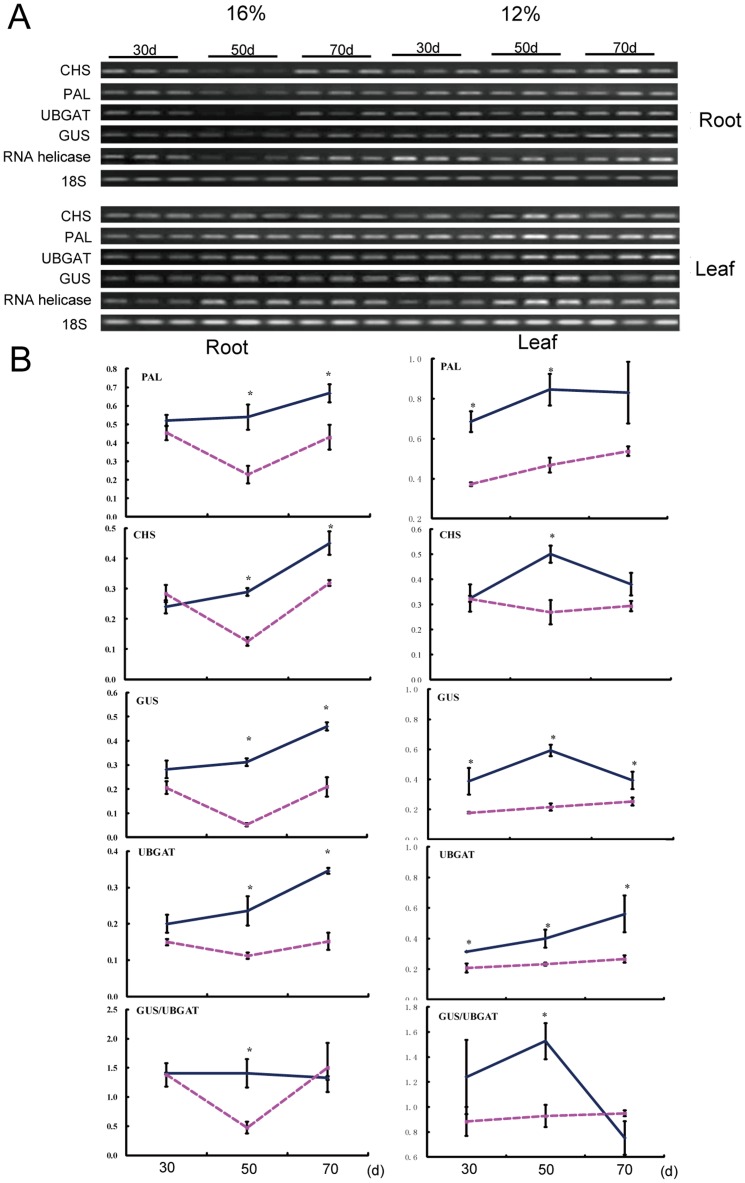
Effects of water deficit on the expression of flavonoid biosynthesis genes in *S. baicalensis*. A, RT-PCR analysis of the transcript levels of interested genes; B, Quantification of the RT-PCR results. The broken line is for the 16% SWC control and the solid line is for the 12% SWC water stress treatment. Vertical bars indicate the standard deviation of three biological replicates. Asterisks indicate a significant difference at the *P*<0.05 level.

We further to investigate whether water deficit affected the expression of the genes involved in flavonoid biosynthesis. Baicalin and baicalein are synthesized via the phenylpropanoid pathway by the activities of several enzymes, including PAL, cinnamate 4-hydroxylase, 4-coumarate:CoA ligase, CHS and chalcone isomerase. By blasting in GenBank with the known gene sequence of Arabidopsis, we found the *S. baicalensis* EST sequences encoding for *PAL* (EF501766), *CHS* (AB008748), *UBGAT* (EF512580) and *GUS* (AB040072). The specific primers for these genes were designed and semiquantitative RT-PCR was performed (The primers were shown in Table S1). The results showed that water deficit increased the expression of *PAL*, *CHS*, *GUS* and *UBGAT* both in leaves and roots (Figure 2), and the expression pattern were similar in leaves and roots. Transcript level of *GUS* was increased to a greater extent than those of *UBGAT*. In further investigations, we were more interested in what happened in *S. baicalensis* root, because root is used in Chinese medicine and contains the highest concentrations of flavonoids than other organs.

### Proteome changes in *S. baicalensis* roots under water deficit

To further elucidate the mechanisms that stimulated the accumulation of flavoinds in the roots of *S. baicalensis* under water deficit, the proteome change was investigated using two-dimensional gel electrophoresis (2-DE) method. For high reproducibility and low background on gels, a silver staining approach was used to detect protein spots on 2-DE gels, and the 2-D protein patterns was shown in Figure S1. These 2-DE gels averaged around 1300 spots/gel and more than 600 spots overlapped on these gels. The protein profile was highly reproducible among three replicate samples.

The protein spots showing a significant change in volume were selected; and a total of 29 spots showed altered expression patterns following water deficit. These 29 spots were excised and identified by mass spectrometry (MS) analysis. Only 24 spots could be successfully identified as unique hits in searches of the NCBInr database generated by the BGI and *S. baicalensis* cDNA library using the MASCOT program. Protein identifications were considered significantly when at least three peaks matched the protein. The MS spectrum and the matched peptide fragments of three identified proteins were shown in Figure S2. Among the 24 proteins, 15 proteins such as a putative R2R3-Myb transcription factor, a putative electron transporter, an adenosylhomocyteinase, and a chloroplast heat shock protein 70B were up-regulated, and three proteins were down-regulated by water deficit. The expression patterns of other six proteins varied with sampling time (Table 1; Table S2). The proteins identified from the three sets of 2-DE gels were grouped according to their functions as documented in the EBI (http://www.ebi.ac.uk/InterPro) and NCBI databases. Most of the proteins with differential expression belonged to a group of metabolism-related proteins according to the KO pathway (Figure S3).

**Table 1:** Differentially expressed proteins which have *S. baicalensis* cDNA sequence.

Spot no.	NCBI accession No.	Protein name	Average -fold change^a^			Score	M^b^	C^c^ (%)	NCBI accession No^d^.
			30 d	50 d	70 d				
**Up-regulated protein Spots**									
001	AAO42850	2-oxoglutarate (2OG) and Fe(II)-dependent oxygenase superfamily protein	1.088	3.830	ns^e^	68	5	23	JX068528
004	EEF28373	R2r3-myb transcription factor, putative	ns	1.789	1.879	71	7	26	JX068530
005	EEF33225	Electron transporter, putative	2.954	5.156	ns	77	8	13	JX068531
009	XP_002313027	BEL1-related homeotic protein, putative	ns	1.192	1.731	78	9	21	JX068534
014	NP_193130	Adenosylhomocysteinase/copper ion binding	ns	1.969	ns	88	10	23	JX068535
015	ACJ24804	Chloroplast heat shock protein 70B	ns	1.258	ns	75	7	73	JX068536
020	AAU84988	Anthranilate synthase alpha 1	ns	1.361	ns	78	9	20	JX068542
021	AAT40104	ADH-like UDP-glucose dehydrogenase	ns	1.306	ns	73	9	20	JX068532
023	BAC98579	ATP-dependent RNA-helicase	1.424	1.326	1.891	79	15	19	ADD65372
024	ACO62538	Mitochondrial carrier family	ns	2.058	ns	73	11	33	JX068538
**Down-regulated protein Spots**									
022	EEF43612	d-3-phosphoglycerate dehydrogenase	ns	0.759	ns	72	11	17	JX068537
026	AAF79147	Alpha-tubulin	ns	0.441	ns	100	8	35	JX068539
**Others**									
008	XP_002318651	pantothenate kinase, putative	0.456	1.642	ns	74	12	12	JX068533
027	NP_001142255	Glycosyltransferase, CAZy family GT8	ns	0.294	3.658	72	7	31	JX068540
028	EEF39512	Chitin-inducible gibberellin-responsive protein, putative	4.840	0.000	ns	44	4	10	JX068541

a Spot abundance is expressed as the ratio of intensities of up-regulated or down-regulated proteins between stress and control. -Fold changes had p values<0.05. 30 d, 50 d, and 70 d represent water stress treatment for 30,50,70 d, respectively.

b Number of mass values matched.

c Sequence coverage.

d identified proteins in protein database of *Scutellaria baicalensis* in our group.

e ns indicates no significant change of spot abundance between stress and control.

Our proteome analysis results clearly showed that water deficit affected the expression level of several proteins related to GA or auxin metabolism. The expression of a GA-responsive protein (EEF39512) increased after 30 d and decreased after 50 d. Water deficit increased the expression level of an R2R3-Myb transcription factor (EEF28373), and an anthranilate synthase (AAU84988), which is related to indoleacetic acid (IAA) synthesis (Table 1).

### Water deficit affected flavonoid accumulation by regulating GAs and IAA metabolism

**Figure 3:**
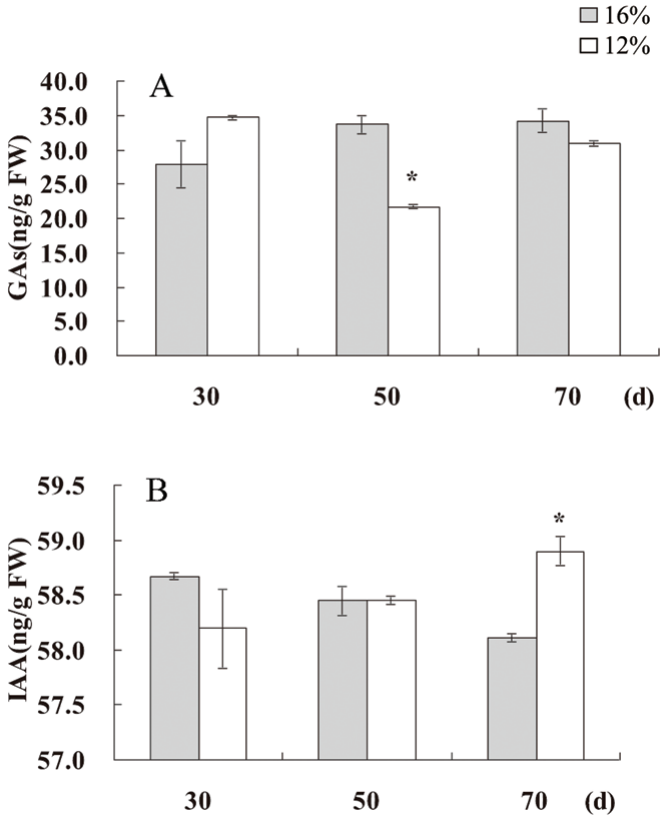
Effects of water deficit on endogenous GAs and IAA. The GA and IAA content of leaves of *S. baicalensis* grown under 16% SWC as a control and 12% SWC as a water deficit treatment. Vertical lines indicate the standard deviation of three biological replicates. Asterisks indicate a significant difference at the *P*<0.05 level.

**Figure 4:**
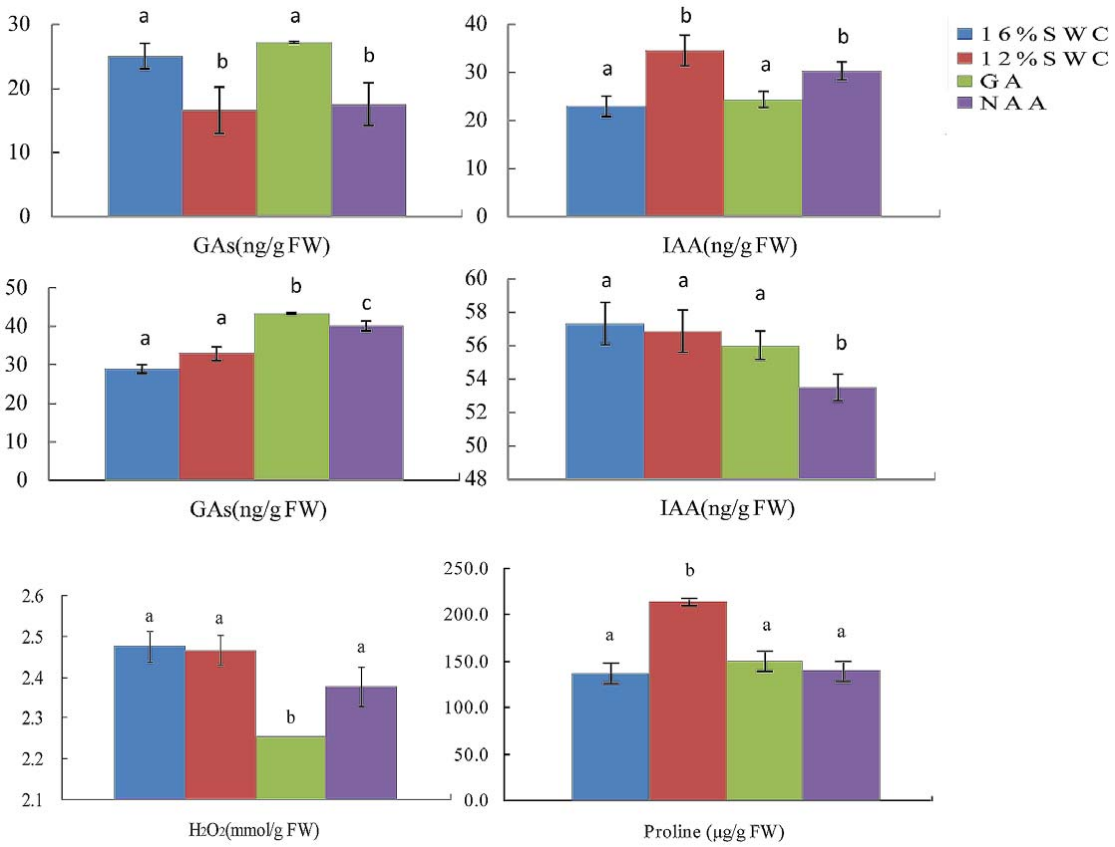
Effects of plant growth regulators on levels of endogenous GAs and IAA in roots (A, B) and leaves (C, D), and on the content of H_2_O_2_ (E) and proline (F) in roots of *S. baicalensis*. Vertical lines indicate the standard deviation of three biological replicates. Asterisks indicate a significant difference at the *P*<0.05 level.

The influence of water deficit on GA- and IAA-related proteins and flavonoid accumulation indicated a possible linkage among water deficit, flavonoid accumulation, and hormone metabolism. To investigate this possibility, we measured endogenous GAs and IAA levels in *S. baicalensis*. Under water deficit conditions, decreased endogenous GAs levels and increased IAA levels were observed in leaves of *S. baicalensis* (Figure 3) and in roots [25], indicating that water deficit affected both GA and IAA metabolism. When exogenous GA_3_ was sprayed on *S. baicalensis* plants grown under water deficit conditions, endogenous GAs levels were increased both in roots and leaves, whereas endogenous IAA levels were decreased in roots (Figure 4). Exogenous GA_3_ also caused a decrease of H_2_O_2_ level (Figure 4). When plants were treated with exogenous auxin NAA, the increased GAs levels and decreased IAA levels in leaves were observed, however GA and IAA levels in roots were not affected. These results indicate that GAs and IAA could affect each other (Figure 4).

**Figure 5:**
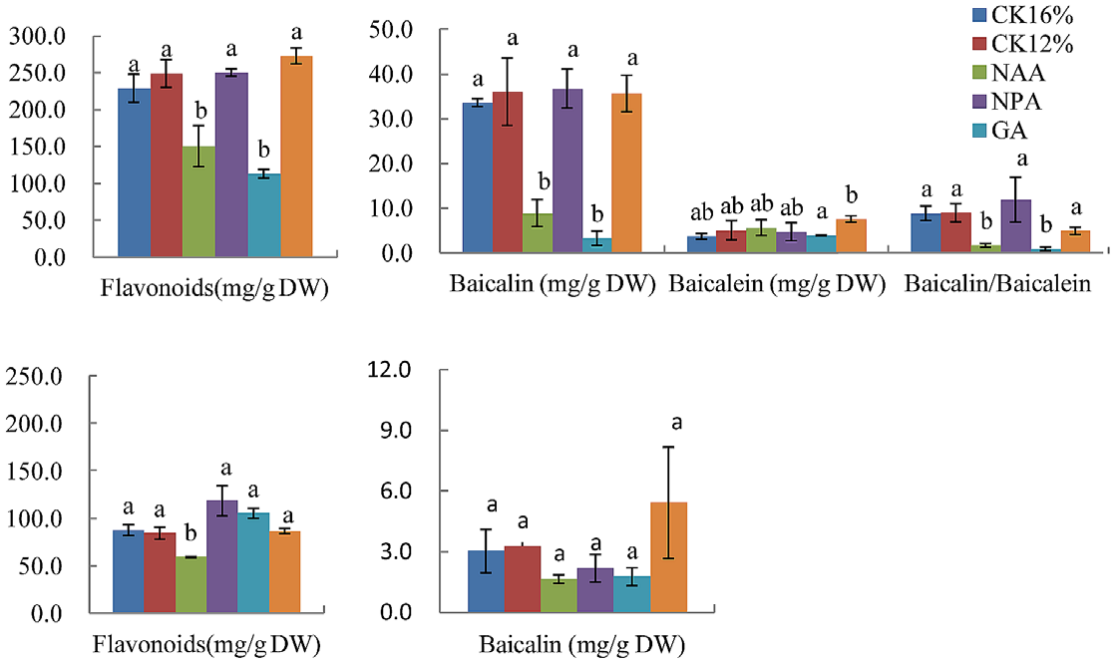
Effects of plant growth regulators on flavonoid levels in *S. baicalensis*. Total flavonoids, baicalin, baicalein, and the ratio of baicalin to baicalein in roots (A, B) and leaves (C, D) of *S. baicalensis*. Vertical bars indicate the standard deviation of three biological replicates. Asterisks indicate a significant difference at the *P*<0.05 level.

To further analyze whether GAs or IAA influence flavonoid metabolism in *S. baicalensis* plants, we examined the flavonoid contents of *S. baicalensis* plants sprayed with exogenous GA_3_, NAA, and their synthesis inhibitors, paclobutrazol and 1-N-naphthylphthalamic acid (NPA), respectively. The content of total flavonoids and baicalin and the ratio of baicalin and baicalein decreased in roots under water stress after application of GA_3_, and these decreases were reversed by application of paclobutrazol (Figure 5). Application of NAA and its synthesis inhibitor 1-N-naphthylphthalamic acid led to results similar to GA_3_ treatment. However application of GA_3_ and its synthesis inhibitor paclobutrazol did not affect total flavonoid content or the baicalin content in leaves under water stress, and NAA treatment decreased flavonoid content (Figure 5). Exogenous IAA treatment led to the similar results as NAA treatment (Figure S4)..When the plants at 16% SWC were sprayed with GA_3_ or NAA, the contents of total flavonoids and baicalin were decreased and baicalein were increased in roots, and these decreases were reversed by application of paclobutrazol and NPA (Figure S5). However, baicalein content was increased by treatment with NAA or GA_3_.

## Discussion

The composition of active compounds in Chinese medicinal plants determines the quality of these herbal medicines [26]. For *S. baicalensis*, the flavonoid content and the ratio of baicalin to baicalein are the determinants. The metabolism of flavonoids follows a complex pathway, and some environmental factors such as temperature, water status, light condition, and nitrogen affect the accumulation of flavonoids [27]–[30]. In a previous study, we showed that low rainfall levels are closely associated with the content of flavonoids, especially of baicalein, in *S. baicalensis* plants from 19 production areas [24], indicating that water status is an important factor affecting flavonoid accumulation in *S. baicalensis*.

Water deficit can initiate a complex of responses in plants at the cellular, physiological, and developmental levels [31]. Water deficit also can enhance flavonoid production in ripening fruit [3] and berries [13]–[14], and in cell suspension cultures of *Glycyrrhiza inflata* Batal [15] and *Ligustrum vulgare* [16]. Our previous study showed that total flavonoid accumulation was accelerated after 50 d of water deficit treatment [25]. Expression levels of *PAL* and *CHS*, two key genes in the biosynthesis of flavonoids, were also elevated by water deficit. These results are consistent with those of Yamaguchi et al. [32], who found that water stress significantly increased the total isoflavonoid content in portions of soybean primary root. However, Vincent et al. [11] found that lignin levels were lower in leaves of plants subjected to water deficit than in those of well-watered plants. We did not find any change in baicalin accumulation with water deficit in leaves of *S. baicalensis*.

The mechanism of water deficit tolerance in *S. baicalensis* and its relation to changes of flavonoids was further examined. The 2-DE analysis combined with MS is a powerful approach to identify the proteins involved in plant drought tolerance. The proteins involved in ROS metabolism, isoflavonoid biosynthesis, control of apoptosis-like cell death, and control of protein degradation in soybean root were affected by water stress [32]. In Arabidopsis, 30% of the transcriptome responds stress and 1008 mRNAs were specifically upregulated by water deficit [33]. In our experiment, water deficit affected four functional categories in *S. baicalensis* including hormone metabolism, carbohydrate metabolism, proteins responsive to stress, and transcript factors.

In our experiment, a total of 29 spots showed altered expression patterns following water deficit treatments, and 24 spots were identified. In this study, we run the MS analysis for the protein identification. MS data may have false discovery compared to MS/MS data, so we identified the protein with at least three match peaks. Among the 24 proteins, 15 proteins were upregulated and three proteins were down-regulated by water deficit. The other six proteins exhibited diverse expression patterns depending on sampling time. In the 24 identified proteins, the expressed level of ATP-dependent RNA-helicase (BAC98579) increased at all times under water deficit conditions compared to the control (Figure S6). The results of semiquantitative RT-PCR analysis showed that transcript pattern was similar to protein expression (Figure S6), confirming the reliability of the 2-DE results. Wehmeyer et al. [34] suggested that heat shock proteins are involved in desiccation tolerance, and Hajheidari et al. [35] reported that two sHSPs were up-regulated under drought stress in sugar beets grown in the field. In this experiment, a chloroplast heat shock protein 70B (ACJ24804) was somewhat up-regulated. In other reports on proteomics analysis of drought stress, many more significantly changed proteins were identified. For example, 58 proteins were identified in *Elymus elongatum* [36] and 49 changed proteins were found in peanut [37]. Fewer significantly changed proteins were identified in our experiment compared with these reports, might due to the fact that the *S. baicalensis* plants adapted to the water deficit treatment (12% SWC) in our experiment, resulting in fewer proteins being affected. In other previous works, the water deficit condition similar to that used in this study led to the increase of detoxifying proteins such as superoxide dismutase and dehydroascorbate reductase in *Elymus elongatum* [36], and proteins relating to biosynthesis of jasmonic acid and ABA signaling [37].

In our proteome analysis, the expression levels of several proteins related to GA or auxin metabolism were affected. The expression of a GA-responsive protein (EEF39512) increased after 30 d, but then decreased after 50 d. Water deficit increased the expression level of an R2R3-Myb transcription factor (EEF28373) and an anthranilate synthase (AAU84988), which is related to IAA synthesis (Table 1). The MYB transcription factors play important roles in the regulation of many secondary metabolites at the transcriptional level, and Arabidopsis R2R3-Myb transcription factors have been reported to play roles in flavonoid biosynthesis in lettuce [38]. Devaiah et al. [39] reported on the role of MYB62 in the regulation of phosphate starvation responses via changes in GA metabolism and signaling. We also detected an increased expression level of a putative R2R3-Myb transcription factor in *S. baicalensis* roots that has high identity with AtMYB113, which is involved in the regulation of anthocyanin biosynthesis, indicating that the R2R3-Myb transcription factor is also involved in flavonoid biosynthesis in *S. baicalensis*.

The effect of water deficit on GA- and IAA-related proteins and flavonoid accumulation prompted us to consider the possible linkage among water deficit, flavonoid accumulation and hormone metabolism. Plant hormones affect the accumulation of secondary metabolites. Decrease of the *CsPAL* expression level and catechin content in response to ABA and GA_3_ treatment has been observed [40]. GA_3_ may inhibit the phenylpropanoid pathway at the level of PAL in *Myrica rubra* [41], pea [42] and carrot [43]. Exogenous ABA application can also increase the expression level of anthocyanin synthesis-related genes and the concentration of anthocyanins in grape berries [20]. Meanwhile, plant flavonoids inhibit auxin transport primarily at the shoot apex and root tip, and flavonoid changes are subsequent to auxin accumulation in some auxin-accumulating tissues [44]. In *S. baicalensis*, the total flavonoid content increased in leaves and did not change significantly in roots [25]. Water deficit decreased the level of endogenous GAs and increased IAA levels in leaves and roots of *S. baicalensis*. Exogenous GA_3_ increased endogenous GAs levels in both roots and leaves of *S. baicalensis* grown under water deficit conditions, and decreased endogenous IAA levels in roots. Exogenous NAA increased GA levels and decreased IAA levels in leaves, but their levels in roots were not affected. These results indicate that GAs and IAA may affect each other. Ribnicky et al. [45] reported that NAA treatment for 4 weeks led to a 50% decrease in the concentration of total IAA in carrot. The reason that exogenous NAA affect endogenous IAA might be due to the decrease of the biosynthesis of endogenous IAA. The levels of total flavonoids and baicalin and the ratio of baicalin to baicalein in roots were decreased under water deficit condition after application of GAs, and these decreases were recovered after application of paclobutrazol. Application of NAA and its synthesis inhibitor NPA produced similar results as GAs and paclobutrazol treatment. However, application of GAs and its synthesis inhibitor paclobutrazol did not affect the levels of total flavonoids and baicalin in leaves under water stress, and IAA treatment decreased flavonoid content. These results clearly show that plant hormones, particularly GAs and IAA, affected flavonoid metabolism, and that water deficit affected flavonoid accumulation by regulating hormone metabolism in *S. baicalensis*.

## Materials and Methods

### Plant material and experimental conditions

The seeds of *S. baicalensis* were obtained from Institute of Chinese Materia Medica, Academy of Chinese Medical Sciences, Beijing, China), sterilized in 0.5% NaOCl for 5 min, then washed 3 times with sterile water, and placed in petri dishes to germinate. The seedlings five days after germination were transferred to individual pots (10 seedlings per pot) containing 500 g dried soil in climate chamber at 25°C with 16 h-light photoperiod under well-water condition. Three months later, pots were weighted at 9 am each day, and the soil water content at saturation was determined experimentally adding a known volume of water to the pots. Plants were irrigated with calculated volume of distilled water to maintain the soil water content at 16% or 12%. The roots and leaves were sampled three times at 30, 50, 70 d of water stress.

GA_3_, NAA and their inhibitor P and NPA (100 mg/L) were spayed every five days for one month on leaves of 3-month-old plants grown at 12% SWC. The roots and leaves were sampled three times. The sample were rinsed three times in distilled water, and then stored at −80°C for further experiments.

### Two-Dimensional Gel Electrophoresis (2-DE)

The *S. baicalensis* root samples from plants exposed to water-stress for 30, 50, and 70 d and control roots were homogenized to powder in liquid nitrogen. Proteins were extracted with phenol according to Wang et al. [46]. Total protein content was estimated according to the method described by Bradford [47].

The proteins (0.2 mg/gel) were mixed with rehydration buffer containing 8 M urea, 2% (w/v) CHAPS, 20 mM DTT, 0.5% (v/v) IPG buffer (pH 4–7), and 0.002% bromophenol blue and rehydrated overnight with IPG strips (18 cm with a linear gradient of pH 4–7). Isoelectric focusing was carried out for 50 kVh using IPGphor (Amersham Biosciences) at 20°C. Subsequently, the IPG strips were treated with reduction and alkylation in SDS-PAGE running buffer at equilibrium. These strips were then loaded and run on 12% acrylamide Laemmli gels (26×20 cm) using the Ettan DALT II system (Amersham Biosciences) with a programmable power control for 0.5 h at 2.5 W per gel, and then at 15 W per gel until the dye front reached the gel bottom. The separated proteins were visualized using silver staining. The 2-DE gels were made in triplicate from six independent protein extractions.

### Image analysis

Images of the stained gels were acquired with an Image Scanner (Amersham Biosciences) in a transmission mode. The gel images were subsequently analyzed by ImageMaster 2D Platinum (Amersham Biosciences). To produce comparable data for quantitative analysis, several key parameters in the image analysis were fixed as the constants, such as smooth at 2, saliency at 300, and Min Area at 100. Based on the analyzed results from the software, the gel images were further analyzed manually, with particular attention paid to checking the differential spots on the gels.

### Tryptic digestion

The gel spots were excised, successively destained, and dehydrated with 50% acetonitrile (ACN) in 25 mM ammonium bicarbonate. The proteins were then reduced with 10 mM DTT in 25 mM ammonium bicarbonate at 56°C for 1 h and alkylated by 55 mM iodoacetamide in 25 mM ammonium bicarbonate in the dark at room temperature for 45 min. Finally, the gel pieces were thoroughly washed with 25 mM ammonium bicarbonate in water/ACN (50/50) solution and completely dried in a SpeedVac Concentrator. Proteins were incubated in 2.5 mL of modified trypsin solution (10 ng/mL in 25 mM ammonium bicarbonate) for 30 min on ice, and 10 mL 25 mM ammonium bicarbonate were added for digestion overnight at 37°C. The digestion reaction was stopped using 1 mL 10% TFA.

### Peptide identification and classification

The digested peptide (10–50 fmoles) was loaded onto a target well of an AnchorChip plate. After drying, 0.1 mL CHCA (4 mg/mL in 70% CH3CN, 0.1% TFA) matrix solution was added to the target well followed by air drying. Subsequently, the sample target was washed twice using 0.1% TFA for desalting. The AnchorChip plate with protein sample was injected into the Bruker AutoFlex MALDI-TOF MS. The mass spectrometer was operated under 19 kV accelerating voltage in reflection mode with an m/z range of 600–4000. The monoisotopic peptide masses obtained from MALDI-TOF MS were analyzed by m/z software. Mass spectra were internally calibrated with peptides arising from trypsin autoproteolysis at m/z = 842.509 and m/z = 2211.105 to reach a typical mass measurement accuracy of 100 ppm.

Monoisotopic peptide masses obtained from MALDI-TOF MS were used to search the NCBInr database generated by the BGI using the MASCOT program (Matrix Science, London, UK), and also search in our *S. baicalensis* cDNA library with 6941 full-length cDNAs, some of which have been submitted to GenBank. The proteins identified from the three sets of 2-DE gels were categorized according to their functions as documented in the EBI (http://www.ebi.ac.uk/InterPro) and NCBI databases. The output from InterProScan and NCBI was analyzed to obtain GO and KO categories of each sequence.

### ROS and Proline measurement

The H_2_O_2_ concentration was measured by monitoring the absorbance of titaniumperoxide complex at 415 nm, following the method of Patterson et al. [48]. Proline was extracted and its concentration determined following the method of Bates et al. [49]. Roots were homogenized with 3% sulfosalicylic acid and centrifuged. The supernatant was treated with acetic acid and acid-ninhydrin, boiled for 1 h, and extracted with toluene. Then, its OD at 520 nm was read. Proline content was expressed as µg/g FW.

### HPLC analysis of flavonoid

To determine flavonoid content, 100 mg powdered material was extracted for 1 h in 1 mL ethyl alcohol. The solution was filtered through a membrane filter (0.2 µm), and flavonoid concentrations were determined using an HPLC system with a 1.0 mL/min flow rate. HPLC was performed on a diamonsil C_18_ column (4.6 mm×250 mm, 5 µm). The detection wavelength was set at 280 nm and the column temperature was maintained at 30°C. The mobile phase consisted of acetonitrile-deionized water-methanoic acid (A; 21∶78∶1, v/v) and acetonitrile-deionized water-methanoic acid (B; 80∶20∶1, v/v). The initial condition was A–B (100∶0, v/v) for 15 min, and this was linearly changed to A–B (87∶13, v/v) at 25 min, to A–B (52∶48, v/v) at 40 min, and to A–B (0∶100, v/v) at 60 min. HPLC grade acetonitrile (E. Merck, Darmstadt, Germany) was used for the HPLC analysis. Peaks were identified using retention time standards from the National Institute for the Control of Pharmaceutical and Biological Products (China). The standard solutions contained 0.208 mg/mL baicalin and 0.602 mg/mL baicalein. The injection volume of the sample solution was 20 µl, and the experiment was repeated six times. The amount of baicalin and baicalein was calculated using the method of Li et al. [50].

### Total flavonoid assay

Total flavonoid content was quantitatively analyzed in samples using the aluminum chlorimetric assay [51], calculated using a standard solution of baicalin. The test was repeated six times.

### ELISA assay for IAA, and GAs

Extraction, purification, and determination of IAA and GAs in leaves by indirect ELISA techniques were performed using the methods of He [52], Yang et al. [53], and Teng et al. [54]. Samples were homogenized in liquid nitrogen and extracted overnight at 4°C in cold 80% (v/v) methanol containing 1 mM butylated hydroxytoluene. The extracts were collected after centrifugation at 10,000 g at 4°C for 20 min, then passed through a C_18_ Sep-Pak cartridge (Waters) and dried in N_2_. The residues were dissolved in PBS (0.01 M, pH 7.4) in order to determine the levels of IAA and GAs. Microtitration plates were coated with IAA- or GAs-ovalbumin conjugates in NaHCO_3_ buffer (50 mM, pH 9.6) and left overnight at 37°C. Ovalbumin solution (10 mg/mL) was added to each well to block nonspecific binding. After incubation for 30 min at 37°C, standard IAA and GA samples and specific monoclonal antibodies (provided by Dr. Baomin Wang, Chinese Agricultural University) were added and incubated for an additional 45 min at 37°C. Horseradish POD-labeled goat anti-rabbit immunoglobulin was then added to each well and incubated for 1 h at 37°C. Finally, the buffered enzyme substrate (*o*-phenylenediamine) was added, and the enzyme reaction was carried out in the dark at 37°C for 15 min and then terminated with 3 M H_2_SO_4_. The absorbance was recorded at 490 nm. Calculations of the enzyme-immunoassay data were performed as described by Weiler et al. [55]. The percentage recovery of each hormone was calculated by adding known amounts of standard hormone to a split extract. Percentage recoveries were all above 90%, and all sample extract dilution curves paralleled the standard curves, indicating the absence of nonspecific inhibitors in the extracts.

### RNA extraction and semiquantitative RT-PCR analysis

For analysis of gene expression by semiquantitative RT-PCR, total RNA was isolated from water-stress roots and control roots using Trizol reagent (Invitrogen). Semiquantitative RT-PCR was carried out for PAL (EF501766), CHS (AB008748), UBGAT(EF512580), GUS(AB040072), RNA helicase (GU561857) and 18S(FJ527609) using the One-Step RT-PCR kit (TakaRa) with primers (Table S1). The 18S gene was chosen as a loading control. The one-step RT-PCR was done as follows: 94°C for 3 min, 31 cycles of 94°C for 30 s, annealing temperature for 40 s, and 72°C for 40 s, and 72°C for 10 min.

## Supporting Information

Table S1 **Primers used in this paper.**
(DOCX)

Table S2 **Differentially expressed proteins identified by MALDI-TOF MS.**
(DOCX)

Figure S1 **Differentially expressed proteins in** ***S. baicalensis*** **roots exposed to water deficit.** Separated proteins from 16% SWC treated roots at 30 d (A), 50 d (C), and 70 d (E) are compared with protein profiles resulting from 12% SWC treated roots at 30 d (B), 50 d (D), and 70 d (F). Marked proteins (T) are named in accordance with Table 1 and Table S2 and were identified by MALDI-TOF MS. The numbers at the top of the gel T denote the pH gradient in the first dimension, while the molecular masses of the 2-D standards are displayed on the right.
(TIF)

Figure S2 **The MS spectrum and the matched peptide fragments of protein spot number 4(A), 20 (B) and 28 (C).**
(TIF)

Figure S3 **Functional classification and distribution of identified proteins.** Unknown proteins include those whose functions have not been described.
(TIF)

Figure S4 **Effects of plant growth regulators on flavonoid levels in** ***S. baicalensis*** **after spay IAA and NAA.** Baicalin (A), baicalein (B) and total flavonoids (C) in roots of *S. baicalensis*. Vertical bars indicate the standard deviation of three biological replicates. Asterisks indicate a significant difference at the *P*<0.05 level.
(TIF)

Figure S5 **Effects of plant growth regulators on flavonoid levels in** ***S. baicalensis*** **under SWC16%.** Total flavonoids, baicalin, and baicalein in roots of *S. baicalensis*. Vertical bars indicate the standard deviation of three biological replicates. Asterisks indicate a significant difference at the *P*<0.05 level.
(TIF)

Figure S6 **Effects of water deficit on the expression of RNA helicase in** ***S. baicalensis*** **.** Protein expression (A) and transcript abundance (B) of RNA helicase in roots of *S. baicalensis* grown under 16% SWC as a control (shaded bars) and 12% SWC as a water deficit treatment (white bars). Vertical lines indicate the standard deviation of three biological replicates. Asterisks indicate a significant difference at the *P*<0.05 level.
(TIF)
