# Supplementary material for: Water Deficit Affected Flavonoid Accumulation by Regulating Hormone Metabolism in Scutellaria baicalensis Georgi Roots
Source: PLoS One. 2012 Oct 15;7(10):e42946. doi: 10.1371/journal.pone.0042946 (PMC3471899; doi:10.1371/journal.pone.0042946)
Supplement: Table S1 — Primers used in this paper. (DOCX) [file pone.0042946.s001.docx]

**Table S1 Primers used in this paper**

| Primer | | Size  (bp) | Annealing temperature(℃) |
| --- | --- | --- | --- |
| PAL-F | - AAGCCCTTACCAAGTTCC | 165 | 65 |
| PAL-R | - GCCCACAGCCTTAGAGTT |  |  |
| CHS-F | 5'-TCCGACTGGAACTCCCTCT-3' | 141 | 60 |
| CHS-R | 5'-TCCCGTATTCGCTAAGCAC-3' |  |  |
| UBGAT -F | 5'-AGCCAA GGAAGCCATAGTCAAC-3' | 174 | 60 |
| UBGAT -R | 5'-CCGAAACAAAGGAAGACGACA-3' |  |  |
| GUS-F | 5'-AGAGCAGTGTGAAGATAAGC-3' | 238 | 58 |
| GUS-R | 5'-CATAGTAGGTCCAGGCAAG-3' |  |  |
| RNA helicase-F | 5'-GGAAAGAGCCAGAGTGTC-3' | 140 | 62 |
| RNA helicase-R | 5'-AGGAGGTCCATTAGGTGA-3' |  |  |
| 18S-F | -CGTTGACTACGTCCCTGCCCTT | 186 | 60 |
| 18S-R | -GTTCACCTACGGAAACCTTGTTACGAC |  |  |
