# Supplementary material for: Water Deficit Affected Flavonoid Accumulation by Regulating Hormone Metabolism in Scutellaria baicalensis Georgi Roots
Source: PLoS One. 2012 Oct 15;7(10):e42946. doi: 10.1371/journal.pone.0042946 (PMC3471899; doi:10.1371/journal.pone.0042946)
Supplement: Table S2 — Differentially expressed proteins identified by MALDI-TOF MS. (DOCX) [file pone.0042946.s002.docx]

**Table S2. Differentially expressed proteins identified by MALDI-TOF MS**
