# Supplementary figures and images for: Water Deficit Affected Flavonoid Accumulation by Regulating Hormone Metabolism in Scutellaria baicalensis Georgi Roots
Source: PLoS One. 2012 Oct 15;7(10):e42946. doi: 10.1371/journal.pone.0042946 (PMC3471899; doi:10.1371/journal.pone.0042946)

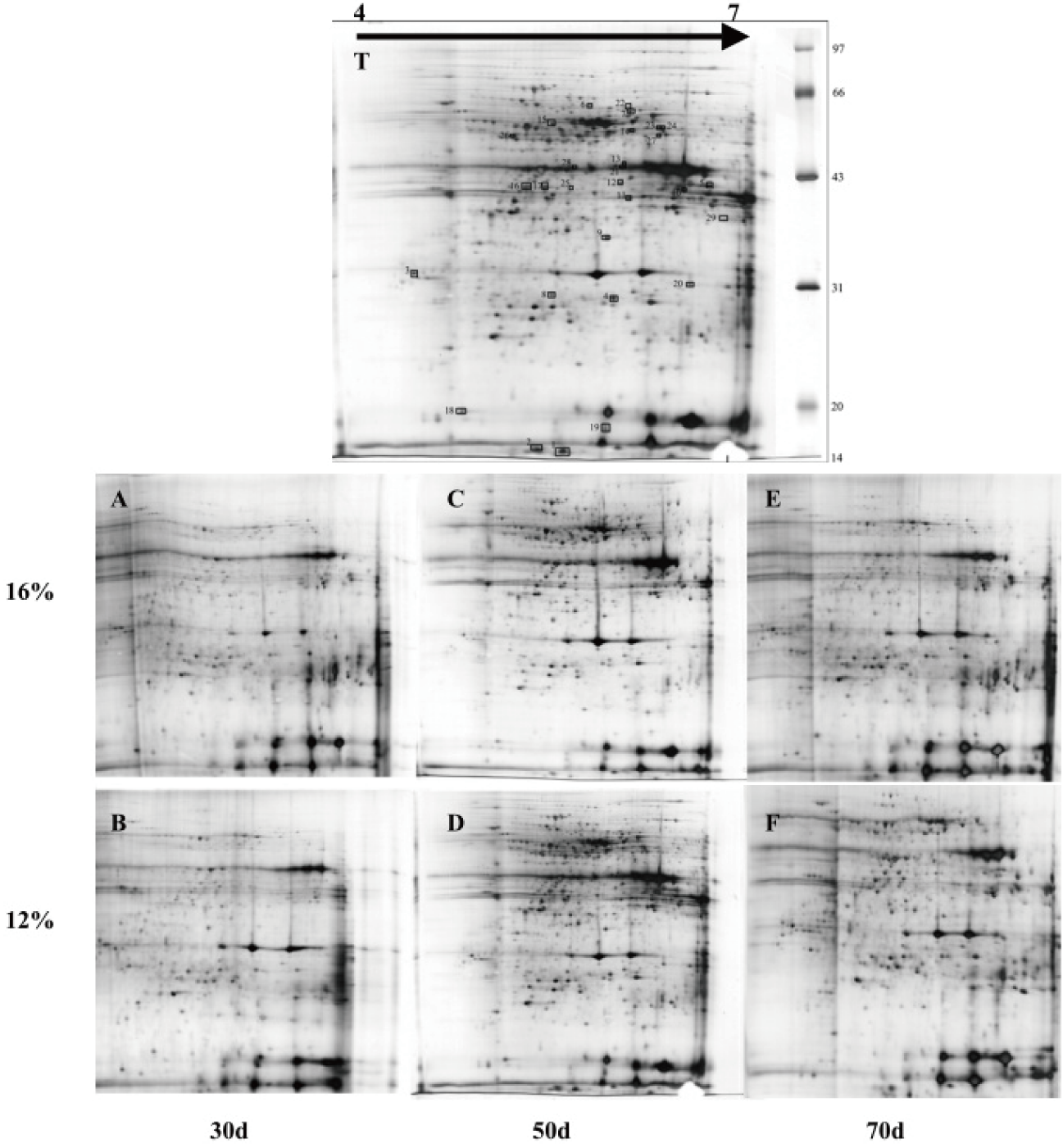

Supplement: Figure S1 — Differentially expressed proteins in S. baicalensis roots exposed to water deficit. Separated proteins from 16% SWC treated roots at 30 d (A), 50 d (C), and 70 d (E) are compared with protein profiles resulting from 12% SWC treated roots at 30 d (B), 50 d (D), and 70 d (F). Marked proteins (T) are named in accordance with Table 1 and Table S2 and were identified by MALDI-TOF MS. The numbers at the top of the gel T denote the pH gradient in the first dimension, while the molecular masses of the 2-D standards are displayed on the right. (TIF) [file pone.0042946.s003.tif]

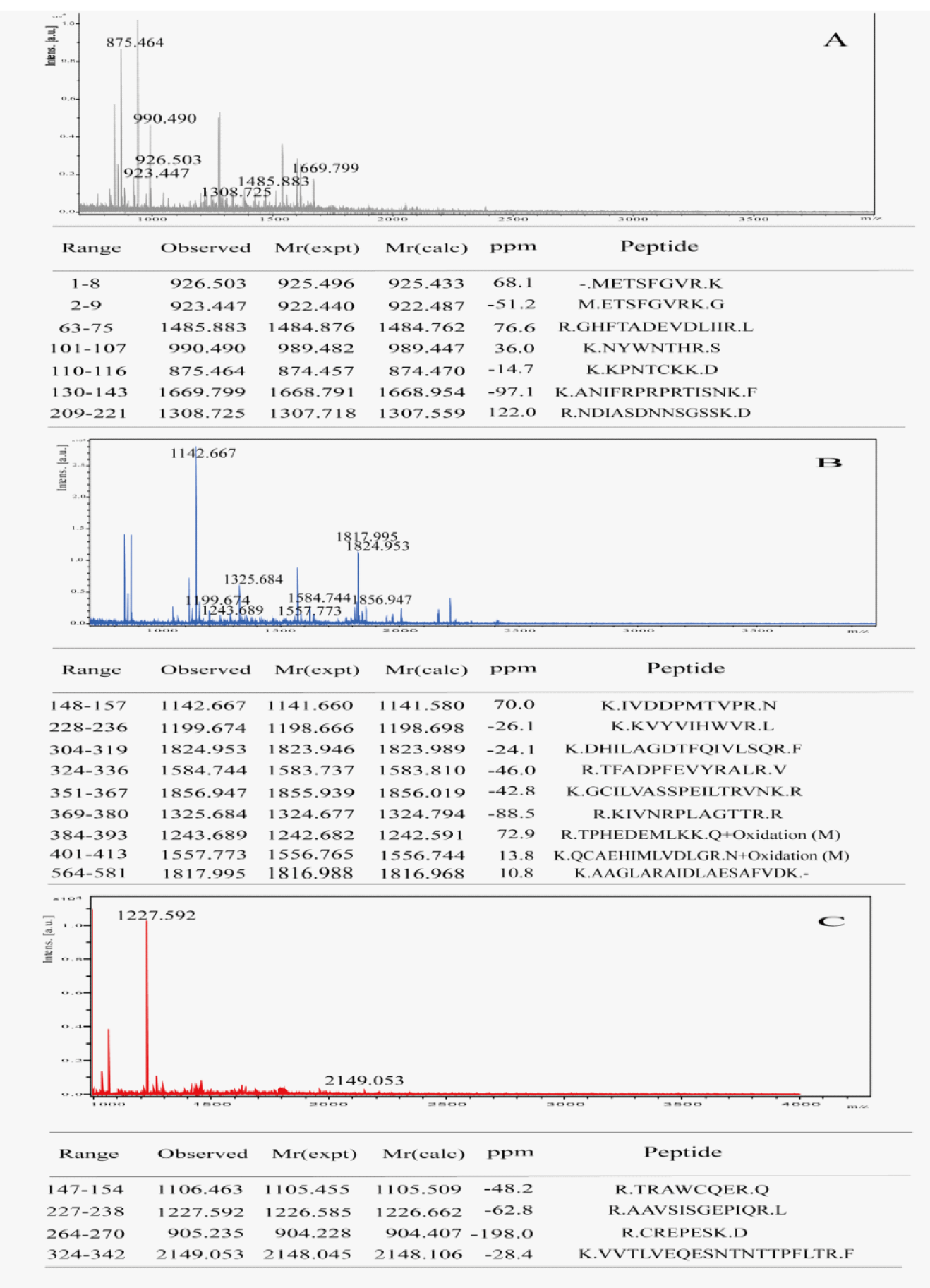

Supplement: Figure S2 — The MS spectrum and the matched peptide fragments of protein spot number 4(A), 20 (B) and 28 (C). (TIF) [file pone.0042946.s004.tif]

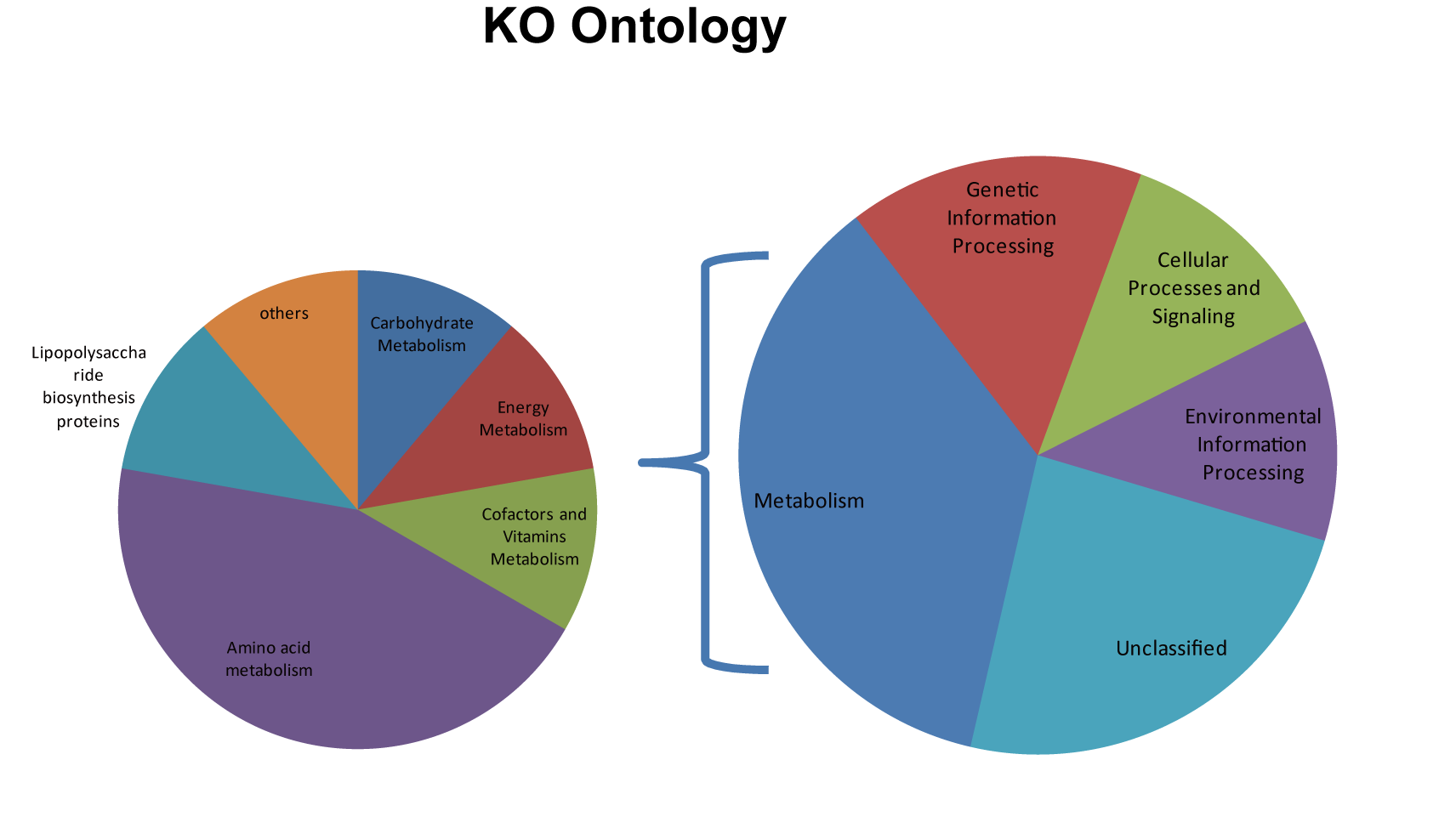

Supplement: Figure S3 — Functional classification and distribution of identified proteins. Unknown proteins include those whose functions have not been described. (TIF) [file pone.0042946.s005.tif]

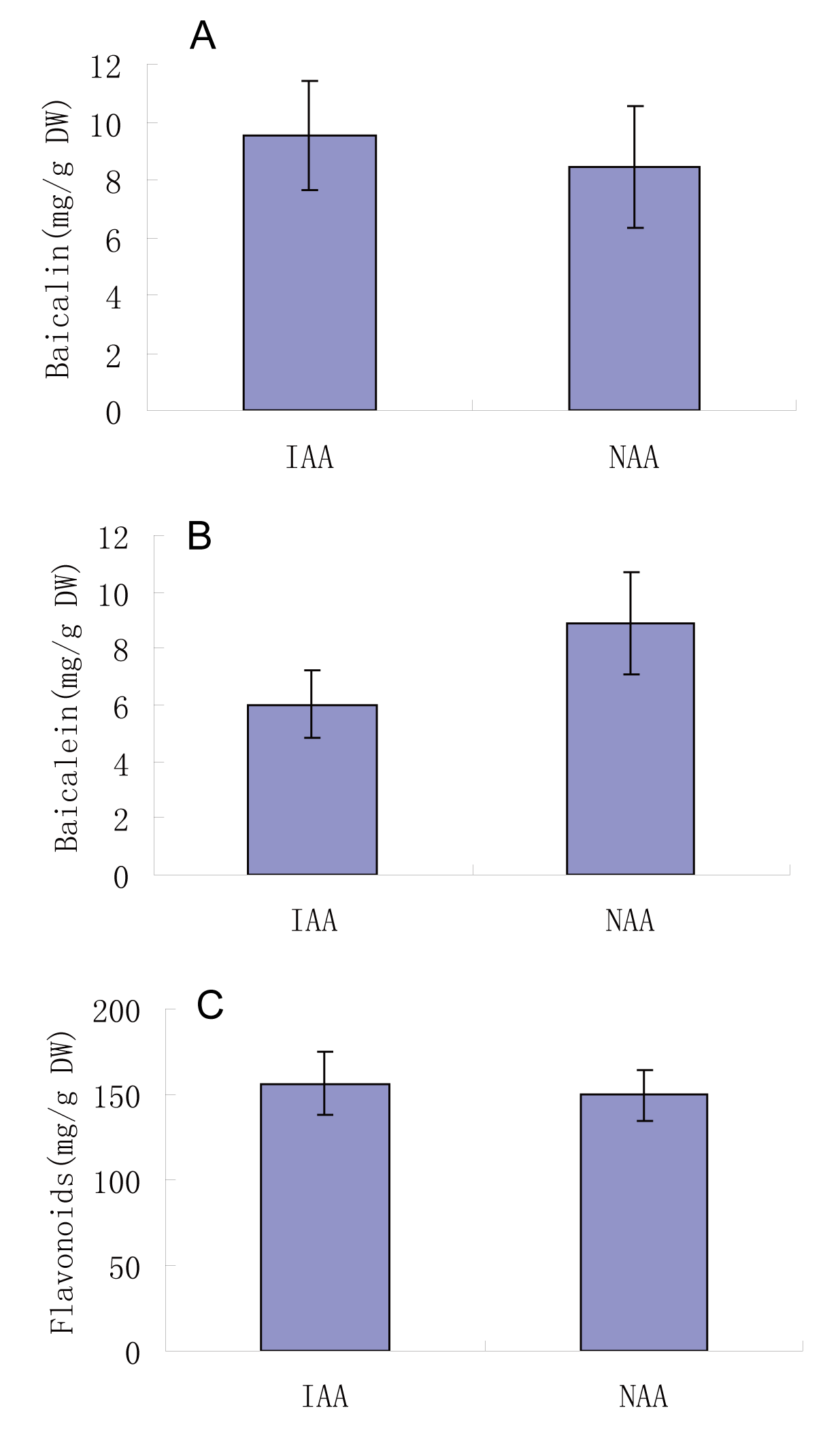

Supplement: Figure S4 — Effects of plant growth regulators on flavonoid levels in S. baicalensis after spay IAA and NAA. Baicalin (A), baicalein (B) and total flavonoids (C) in roots of S. baicalensis. Vertical bars indicate the standard deviation of three biological replicates. Asterisks indicate a significant difference at the P<0.05 level. (TIF) [file pone.0042946.s006.tif]

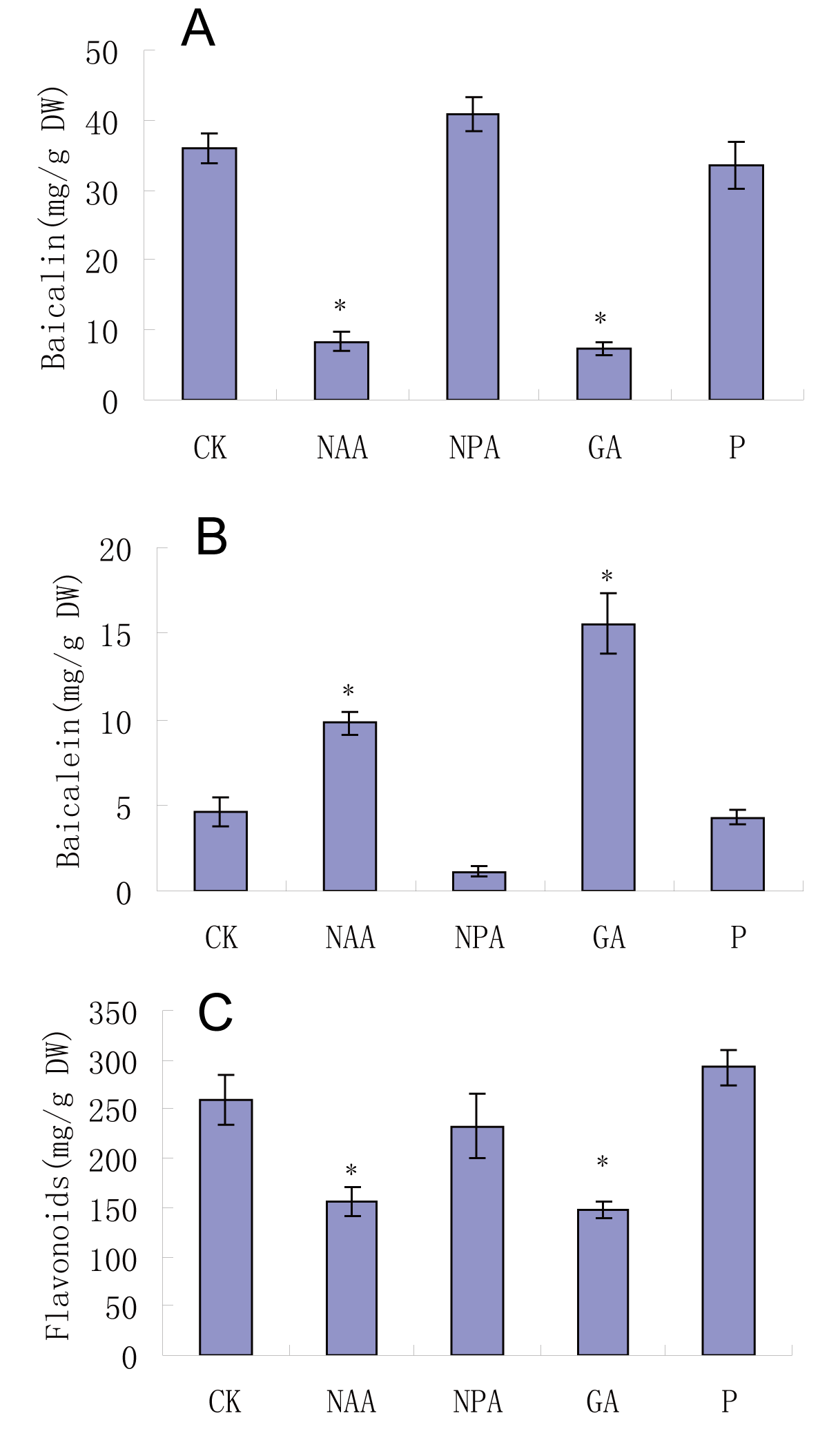

Supplement: Figure S5 — Effects of plant growth regulators on flavonoid levels in S. baicalensis under SWC16%. Total flavonoids, baicalin, and baicalein in roots of S. baicalensis. Vertical bars indicate the standard deviation of three biological replicates. Asterisks indicate a significant difference at the P<0.05 level. (TIF) [file pone.0042946.s007.tif]

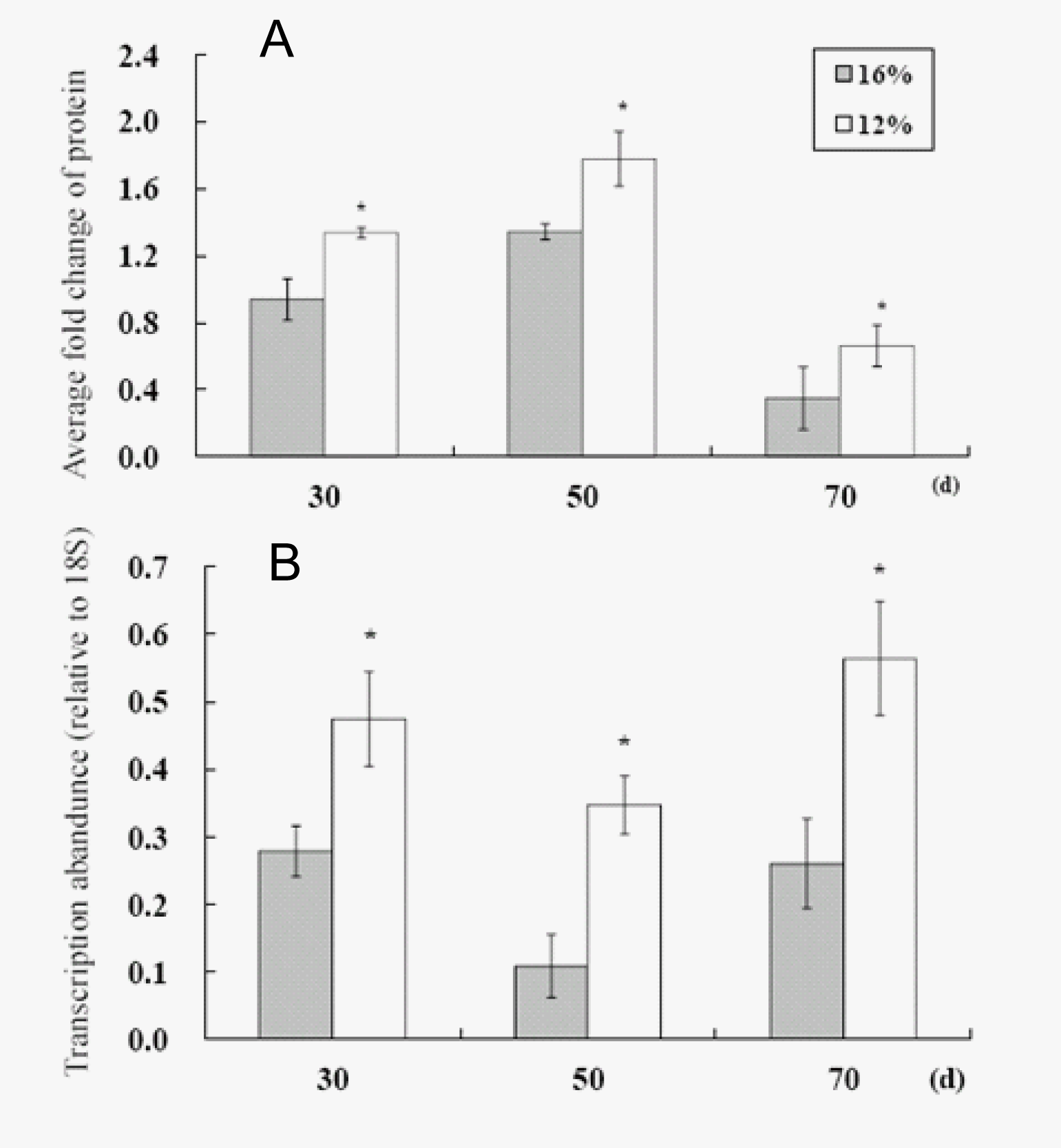

Supplement: Figure S6 — Effects of water deficit on the expression of RNA helicase in S. baicalensis . Protein expression (A) and transcript abundance (B) of RNA helicase in roots of S. baicalensis grown under 16% SWC as a control (shaded bars) and 12% SWC as a water deficit treatment (white bars). Vertical lines indicate the standard deviation of three biological replicates. Asterisks indicate a significant difference at the P<0.05 level. (TIF) [file pone.0042946.s008.tif]
